# A long shelf-life melon created via CRISPR/Cas9 RNP-based *in planta* genome editing

**DOI:** 10.3389/fgeed.2025.1623097

**Published:** 2025-06-18

**Authors:** Kentaro Sasaki, Kaoru Urano, Naozumi Mimida, Satoko Nonaka, Hiroshi Ezura, Ryozo Imai

**Affiliations:** ^1^ Genome-Edited Crop Development Group, Institute of Agrobiological Sciences, National Agriculture and Food Research Organization (NARO), Tsukuba, Japan; ^2^ Sanatech Life Science Co. Ltd., Minato-ku, Tokyo, Japan; ^3^ Tsukuba Plant Innovation Research Center, University of Tsukuba, Tsukuba, Japan; ^4^ Department of Agricultural Sciences, Institute of Life and Environmental Sciences, University of Tsukuba, Tsukuba, Japan

**Keywords:** melon, in planta genome editing, particle bombardment, CRISPR/Cas9 RNP, DNA-free, ethylene

## Abstract

Genome editing in melon (*Cucumis melo* L.) remains a significant challenge due to the inefficiencies associated with conventional cell culture-based transformation methods. In the present study, a novel *in planta* Particle Bombardment (iPB) approach was developed to enable DNA-free genome editing in melon without the need for cell culture. CRISPR/Cas9 ribonucleoproteins (RNPs) were coated onto gold particles and delivered directly into shoot apical meristem tissue, which harbors potential germline cells, via particle bombardment. This method was applied to enhance fruit shelf-life by targeting an ethylene biosynthesis gene (*CmACO1*). The resulting *cmaco1* mutant demonstrated a significantly extended shelf-life, attributable to reduced ethylene production during fruit ripening. This delayed ripening phenotype was reversed upon treatment with exogenous ethylene, confirming the functional impact of *CmACO1* disruption. Because this strategy bypasses cell culture, the iPB-RNP method offers a solution to common limitations in genome editing, such as genotype dependence and somaclonal variation. Consequently, this technique holds substantial promise for advancing commercial melon breeding efforts and may be broadly applicable to other species within the Cucurbitaceae family.

## 1 Introduction

Melon (*Cucumis melo* L.) is a widely consumed fruit across the globe. While conventional breeding has contributed significantly to improving the quality and productivity of melon cultivars, there remains an urgent need for rapid and diversified genetic enhancements. Genome editing is regarded as a promising tool to meet these demands. However, most current genome editing protocols rely on genetic transformation and cell culture, yet melon presents inherent challenges due to its low transformation efficiency, often resulting in the generation of false-positive regenerated plants (escapes) (Shirazi Parsa et al., 2023). Moreover, melon is susceptible to ploidy alterations during cell culture, which can adversely affect morphology of regenerated plants (Ezura et al., 1992; Ayub et al., 1996; Liu et al., 2022; Nonaka et al., 2023). Consequently, the development of an efficient and reliable genome editing system for melon genetic improvement is imperative.

Previous studies have demonstrated a transgene-free genome editing method known as *in planta* particle bombardment-ribonucleoprotein (iPB-RNP), which has been successfully applied in wheat, barley, and soybean (Kumagai et al., 2022; Kuwabara et al., 2024; Tezuka et al., 2024). This technique involves the direct delivery of CRISPR/Cas9 ribonucleoproteins into the shoot apical meristem (SAM), targeting subepidermal L2 cells that serve as potential germline cells (Goldberg et al., 1993). As the iPB-RNP method circumvents the need for cell culture and plant regeneration, it offers considerable potential for application in recalcitrant crop species, including melon.

In the present study, an efficient genome editing platform for melon was established using the iPB-RNP method. CRISPR/Cas9 RNPs successfully induced targeted genome editing in E_0_ plants (the first generation of genome-edited individuals), with a subset of the edited alleles transmitted to the next-generation. Targeting an ethylene biosynthesis gene using this platform enabled the generation of melon with extended shelf-life, wherein fruit ripening can be modulated by exogenous ethylene application.

## 2 Materials and methods

### 2.1 Plant materials

Seeds of the cultivated melon *Cucumis melo* L. var. *reticulatus* (accession “Earl’s Favourite Harukei-3”) were obtained from the GenBank of the National Agriculture and Food Research Organization (NARO), Japan. Fruits of *Harukei-3* and the *cmaco1* mutant were grown under greenhouse conditions and harvested at 45–47 days after pollination for subsequent analyses.

### 2.2 Preparation of SAMs

Mature melon seeds were imbibed at 25°C for 20 h. One cotyledon covering the shoot apical meristem (SAM) was removed from the germinated embryo using tweezers under a stereomicroscope. The embryos were placed upright in Petri dishes containing Murashige and Skoog (MS) basal medium supplemented with sucrose (30 g/L), 2-(N-morpholino) ethanesulfonic acid (MES) monohydrate (0.98 g/L, pH 5.8), a plant preservative mixture (3%; Nacalai Tesque, Japan), and phytagel (7.0 g/L; Sigma-Aldrich, United States). Approximately twenty embryos were placed per dish for subsequent particle bombardment.

### 2.3 Biolistic delivery of GFP plasmids

GFP plasmids (CaMV35S-sGFP(S65T)-NOS3′) (Chiu et al., 1996) were introduced into melon SAMs by particle bombardment as described previously (Hamada et al., 2017). Briefly, 5 µg of plasmid DNA was mixed with 5 µL of 0.6 µm gold particles (Bio-Rad, United States; 40 mg/mL), 10 µL of 0.1 M spermidine, and 25 µL of 2.5 M CaCl_2_, in a final volume of 44 µL. After incubation at room temperature for 10 min, the DNA-coated particles were centrifuged (9,100 × g for 1 s), and the supernatant was discarded. The pellet was washed with 70 µL of 70% ethanol, then resuspended in 30 µL of 99.5% ethanol and sonicated for 1 s immediately prior to use. Aliquots (6 µL) were applied to macrocarrier membranes (Bio-Rad, United States) and allowed to dry in a clean bench. Bombardments were conducted using a PDS-1000/He™ particle delivery system (Bio-Rad, United States) under a vacuum of 27 inches Hg and helium pressure of 1,350 psi. Each plate was bombarded four times.

### 2.4 Observation of GFP fluorescence

Fluorescence in bombarded tissues was observed using an MZFLIII fluorescence stereomicroscope (Leica, Germany) equipped with a GFP filter set (excitation: 470/40 nm; emission: 525/50 nm).

### 2.5 Preparation of Cas9 protein and guide RNA

Recombinant *Streptococcus pyogenes* Cas9 protein was purified from *Escherichia coli* as previously reported (Kunitake et al., 2019). Guide RNAs for genome editing were obtained from FASMAC (Kanagawa, Japan) and Integrated DNA Technologies (Coralville, IA, United States). For CAPS analysis, guide RNAs were synthesized by *in vitro* transcription using the GeneArt™ Precision gRNA Synthesis Kit (Thermo Fisher Scientific, United States). Templates for *in vitro* transcription were designed and amplified using specific primers (Supplementary Table S1) following the manufacturer’s instructions.

### 2.6 Biolistic delivery of RNPs

Purified Cas9 protein and guide RNAs targeting *CmGAD1* or *CmACO1* (Supplementary Tables S2, S3) were mixed with 0.6 µm gold particles (Bio-Rad, United States) and delivered into SAMs as described in a previous study (Kumagai et al., 2022). CRISPR/Cas9 RNPs were assembled by incubating SpCas9 protein (250 pmol) with guide RNAs—either chemically synthesized crRNA and tracrRNA targeting *CmGAD1* (250 pmol each) or chemically synthesized sgRNA targeting *CmACO1* (700 pmol)—in 20 µL of CutSmart® buffer (New England Biolabs, United States) for 10 min at room temperature. Following the addition of 5 µL of TransIT®-LT1 transfection reagent (Takara, Japan), the mixture was incubated for 5 min at room temperature. This RNP mixture was then combined with 25 µL of gold particle solution (40 mg/mL) and incubated on ice for 10 min. After centrifugation at 2,300 × g for 1 s, the pellet was resuspended in nuclease-free water. The RNP-coated particles were spread onto a hydrophilic film (3M, United States) and air-dried for 15 min at room temperature. Bombardments were conducted using a PDS-1000/He™ device (Bio-Rad, United States) under a vacuum of 27 inches Hg and helium pressure of 1,350 psi. Each plate received four bombardments.

### 2.7 Plant growth condition after bombardment

Bombarded embryos were transferred to fresh MS plates and incubated in darkness at 25°C for 3 days. The plates were then moved to a growth chamber under long-day conditions (16 h light/8 h dark, 25°C) and cultured for 2–3 weeks, until healthy leaves and roots develop. The seedlings were subsequently transplanted into pots and grown in a phytotron under long-day conditions (16 h light/8 h dark, 22°C). After screening for CRISPR/Cas9-induced mutations, positive E_0_ plants were grown in a greenhouse. To obtain seeds, female flowers were hand-pollinated using male anthers from the same plant. One fruit was retained per plant.

### 2.8 Cleaved amplified polymorphic sequences (CAPS) analysis

Genomic DNA was extracted from the sixth leaf of E_0_ plants and the first leaf of E_1_ progeny. PCR amplification was performed using PrimeSTAR^®^ GXL DNA Polymerase (TaKaRa, Japan) with gene-specific primers (Supplementary Table S1) in a 20 µL reaction containing genomic DNA. Amplification was carried out for 30 cycles (98°C for 10 s, 60°C for 15 s, 68°C for 1 min) using a thermocycler. A 5 µL aliquot of each PCR product was digested with SpCas9 (600 ng) and in vitro-transcribed sgRNA (300 ng) in 15 µL of reaction buffer containing 20 mM HEPES-NaOH (pH 7.5), 100 mM KCl, 2 mM MgCl_2_, 1 mM DTT, and 5% glycerol (Nishimasu et al., 2018). Digested products were analyzed by agarose gel electrophoresis. Undigested bands were excised, purified, and cloned into the pCR-BluntII-TOPO vector (Thermo Fisher Scientific, United States) for sequencing.

### 2.9 Ethylene measurement from fruit

To quantify ethylene production, each fruit was placed in a 17-L airtight acrylic chamber for 3 h at 25°C. A 1-mL headspace gas sample was collected and injected into a GC-8A gas chromatograph (SHIMADZU, Japan) equipped with a Porapak Q50/80 alumina column (Shinwa, Japan) and a flame-ionization detector, following a previously described protocol (Ohtsubo et al., 1999). Ethylene production was expressed as nl h^-1^ (g fresh weight)^−1^.

### 2.10 Ethylene treatment

Following harvest, fruits were stored in the dark at 20°C for 7 days prior to ethylene exposure. For treatment, fruits were placed in 30-L containers with 400 ppm ethylene at 20°C for 24 h. After the treatment period, fruits were transferred to ambient air (ethylene-free conditions) and stored for an additional 3 days.

### 2.11 Measurement of extractable juice content and flesh firmness

Juice extraction was performed following a previously described method (Lill and Van Der Mespel, 1988). From a horizontal cross-section of the fruit, six tissue cubes (10 mm × 10 mm × 10 mm) were collected from regions 10 mm inward from the epicarp. Each cube was then quartered into smaller segments (approximately 5 mm × 5 mm × 10 mm) and placed into a 5-mL syringe (Terumo, Japan). The tissue was compressed into a 2-mL tube, and the total weight was recorded. After centrifugation at 12,000 × g for 5 min, the supernatant (juice) was removed and weighed. The juice extraction rate was calculated by dividing the juice weight by the initial tissue weight. Flesh firmness was assessed on the opposite side of the fruit from where the juice sample was taken. Measurements were performed at eight points per fruit using an FT011 penetrometer fitted with an 8-mm diameter probe (Italtest, Italy), and values were expressed in newtons (N), following the previously described procedure (Tatsuki et al., 2013).

### 2.12 RNA extraction for expression analysis

Mesocarp and epicarp tissues were collected at various ripening stages or after ethylene treatment, cut into ∼5-mm cubes, and stored at −80°C until RNA extraction. Total RNA was isolated using RNA-suisui S (Rizo Inc., Japan) followed by purification with the RNeasy Plant Mini Kit (Qiagen, Germany), according to the manufacturers’ instructions.

### 2.13 Quantitative RT-PCR

cDNA synthesis was performed using the SuperScript IV VILO Master Mix (Thermo Fisher Scientific, United States). Quantitative RT-PCR was carried out using TB Green Premix Ex Taq II (TaKaRa, Japan) and the AriaMx Real-Time PCR System (Agilent Technologies, United States). Primers used for expression analysis are listed in Supplementary Table S1.

### 2.14 Accession numbers

Sequence data referenced in this study can be found in the Melonet-DB (https://melonet-db.dna.affrc.go.jp/ or https://gene.melonet-db.jp) under the following accession numbers: *CmGAD1* (MELO3C001938.jh1), *CmACO1* (MELO3C014437.jh1), *CmACO2* (MELO3C004619.jh1), *CmACO3* (MELO3C007425.jh1), *CmACO4* (MELO3C010508.jh1), *CmACO5* (MELO3C019735.jh1), *CmPG1* (MELO3C015128.jh1), *CmPG2* (MELO3C016494.jh1), and *CmADP* (MELO3C023630.jh1).

## 3 Results and discussion

**FIGURE 1:**
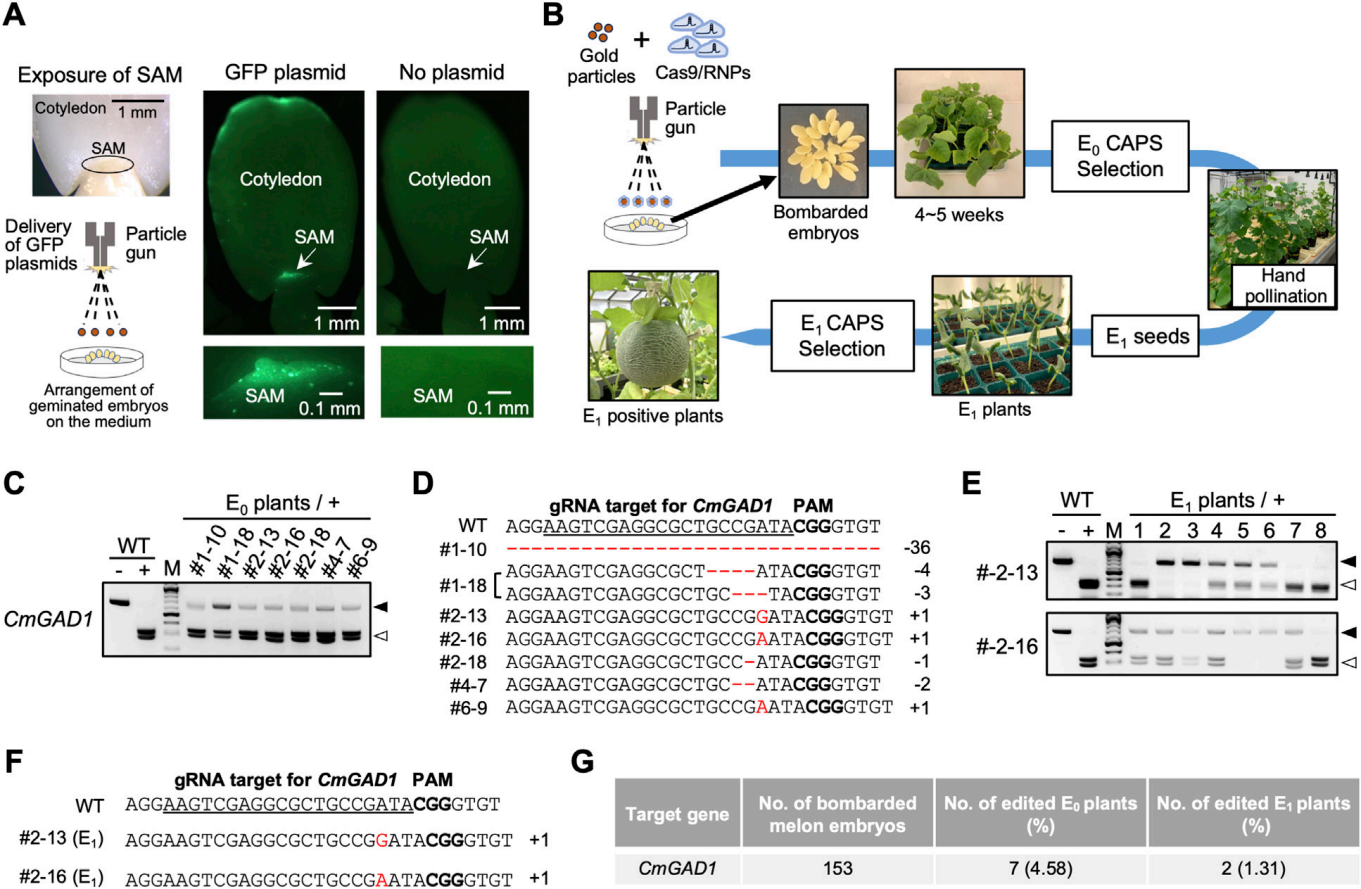
*In planta* RNP-mediated genome editing in melon. **(A)** Microprojectile-mediated transfer of a GFP expression plasmid into melon SAMs. **(B)** Schematic outline of the iPB-RNP method for genome editing in melon. **(C)** CAPS analysis of E_0_ plants carrying mutations at the targeted *CmGAD1* locus. “–” and “+” denote reactions without and with Cas9 RNP digestion, respectively. Black and white triangles indicate undigested and digested bands following Cas9 RNP treatment, respectively. WT, wild-type. **(D)** Alignment of CRISPR/Cas9 target sequences in positive E_0_ plants with the WT sequence. Insertions and deletions are indicated in red. **(E)** Gene-specific CAPS analysis of E_1_ progeny derived from two E_0_ plants (#2–13 and #2–16). Symbol definitions are the same as in panel C. **(F)** Alignment of CRISPR/Cas9 target sequences in positive E_1_ plants with the WT sequence. Insertions are highlighted in red. **(G)** Summary of the genome editing experiment targeting *CmGAD1*. Genome editing efficiency was calculated based on the number of bombarded embryos.

To assess the feasibility of genome editing in melon via iPB-RNP, the efficiency of gold particle delivery into meristematic cells was first evaluated. Following 20 h of seed imbibition, one cotyledon was removed from germinated seeds (cv. *Harukei-3*) to expose the shoot apical meristem (SAM), and the remaining part of each seed was placed on an agar plate with the exposed SAM facing upward (Figure 1A). Gold particles coated with a GFP-expressing vector were then bombarded onto the SAMs (Figure 1A). After 16 h in darkness, GFP fluorescence was observed on the SAM surface in nearly all bombarded seeds, in contrast to untreated controls (Figure 1A; Supplementary Figure S1), consistent with previous observations in wheat and soybean (Hamada et al., 2017; Kuwabara et al., 2024). These results confirmed that the preparation of SAMs and the particle bombardment conditions were suitable for iPB application in melon.

To implement the iPB-RNP method in melon, *CmGAD1*, a glutamate decarboxylase gene (You et al., 2024), was selected as the initial genome editing target. Pre-assembled SpCas9 ribonucleoproteins (RNPs) targeting *CmGAD1* (Supplementary Figure S2) were coated onto gold particles and delivered into the SAMs of germinated seeds. Plants regenerated from the bombarded seeds were analyzed after 4–5 weeks, and their sixth leaf was subjected to cleaved amplified polymorphic sequences (CAPS) analysis to detect mutations (Figure 1B). Seven of the 153 plants initially bombarded exhibited undigested bands in the CAPS assay (Figure 1C). Subsequent sequencing of these undigested bands confirmed the presence of insertion or deletion mutations involving single base pairs (Figure 1D). The editing efficiency in the primary (E_0_) generation was 4.58% (7/153) (Figure 1G). As the E_0_ mutants were chimeric, containing a mixture of edited and non-edited cells, seeds were collected from these plants and the progeny (E_1_ generation) was analyzed to confirm stable inheritance of mutations. CAPS analysis detected mutant *CmGAD1* alleles in two of the seven E_0_ progenitor plants (Figures 1E,G). Among these, plants #2–13_2, #2–13_3, #2–16_5, and #2–16_6 were identified as homozygous mutants for *CmGAD1* (Figure 1E), and sequencing confirmed that the E_1_ mutations were consistent with those observed in the E_0_ generation (Figures 1D,F). The overall editing efficiency of iPB-RNP for *CmGAD1* was 1.31% (2/153 bombarded SAMs) (Figure 1G), which is comparable to editing efficiencies previously reported in wheat, barley, and soybean (Kumagai et al., 2022; Kuwabara et al., 2024; Tezuka et al., 2024).

**FIGURE 2:**
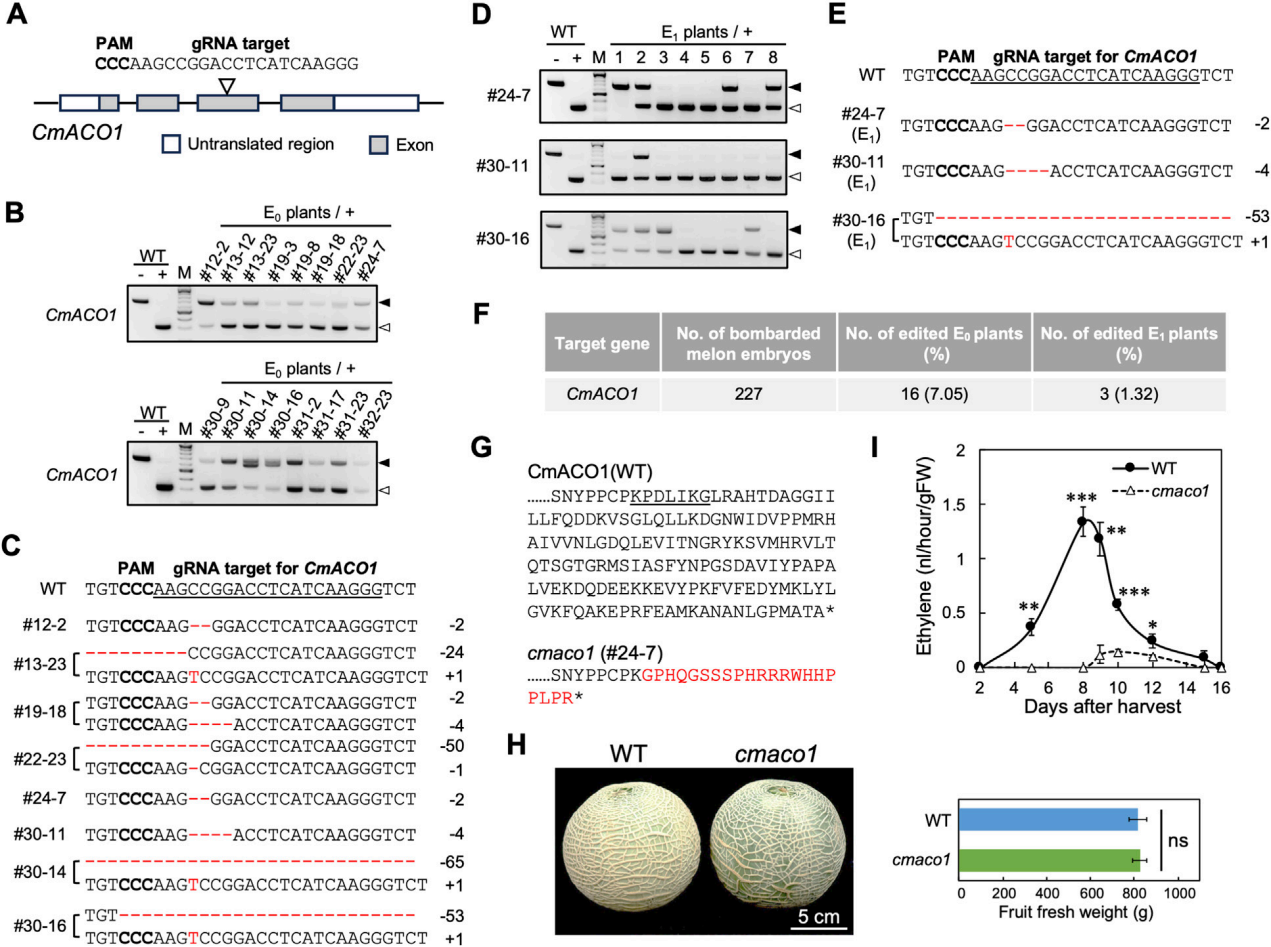
Introduction of *cmaco1* mutations in melon. **(A)** Schematic representation of the *CmACO1* gene with gRNA design. **(B)** CAPS analysis of positive E_0_ plants carrying mutations at the targeted *CmACO1* locus. “–” and “+” denote digestion without and with Cas9 RNPs, respectively. Black and white triangles indicate undigested and digested bands following Cas9 RNP treatment, respectively. WT, wild-type. **(C)** The CRISPR/Cas9 target sequences of selected positive E_0_ plants in B are aligned with that of WT. Insertions and deletions are indicated in red. **(D)** A gene-specific CAPS analysis of E_1_ plants derived from three positive E_0_ plants (#24–7, #30–11, and #30–16). The meaning of symbols is the same as in B. **(E)** The CRISPR/Cas9 target sequences of the positive E_1_ plants are aligned with that of WT. The meaning of symbols is the same as in C. **(F)** Summary of the genome editing experiments targeting *CmACO1*. **(G)** Amino acid sequences of *CmACO1* in wild-type (WT) and the *cmaco1* mutant. Letters with underlines represent the CRISPR/Cas9 target site. Altered sequences are shown in red. An asterisk indicates a stop codon. **(H)** Fruit appearance of WT and *cmaco1* mutants after harvest (11 DAH), and fruit fresh weight of WT and *cmaco1* mutants at 45 days after pollination. Data are presented as means ± SD (n = 12). Student’s t-test was used for statistical comparison between WT and *cmaco1* fruit. ns, not significantly different. **(I)** Ethylene production in fruits of WT and *cmaco1* mutants after harvest. Data are presented as means ± SD (n = 4). P < 0.05; *P < 0.01; **P < 0.001; Student’s t-test compared to *cmaco1* mutant.

Establishment of the iPB-RNP method in melon enabled the generation of mutants with an extended shelf-life, a highly desirable trait for reducing postharvest losses and expanding marketability. Because the gaseous phytohormone ethylene plays a central role in fruit ripening, *CmACO1*, a key gene involved in the final step of ethylene biosynthesis in melon, was selected as the target (Nonaka et al., 2023). A guide RNA was designed and synthesized to target *CmACO1* (Figure 2A). Assembled Cas9 RNPs were delivered into SAMs via iPB-RNP, and resulting mutants were screened using CAPS analysis, as previously described. In total, 16 E_0_ plants carried mutations, and heritable mutations were detected in three plants (#24–7, #30–11, and #30–16) (Figures 2B–E). Ultimately, three genome-edited lines were obtained from 227 bombarded SAMs, corresponding to a 1.32% editing efficiency (Figure 2F). CAPS analysis in the E_1_ generation indicated that the #24–7_1 plant was a homozygous mutant, while the #30–11 and #30–16 plants yielded only heterozygous progeny (Figure 2D).

To investigate the phenotypic consequences of *CmACO1* disruption, fruit from the wild-type (WT) and the *cmaco1* homozygous mutant (#24–7) were compared. The #24–7 line carried a two-base deletion within the *CmACO1* coding region, indicating a likely loss-of-function mutation (Figures 2E,G). Fruit size and shape in the *cmaco1* mutant were similar to those of WT, and no significant difference in fresh fruit weight was observed between the two genotypes (Figure 2H). Eleven days after harvest (11 DAH), the epicarp of WT fruit had turned from green to cream-yellow, indicative of ripening, whereas the *cmaco1* mutant fruit remained green (Figure 2H). To confirm whether the delayed ripening phenotype was attributable to reduced ethylene production, ethylene emission was measured post-harvest. WT fruit exhibited a sharp ethylene peak at 8 DAH, whereas *cmaco1* fruit maintained significantly lower ethylene levels up to 16 DAH (Figure 2I). In a previous study, a *cmaco1* mutant generated via Agrobacterium-mediated genome editing showed reduced fruit size and flattened shape, attributed to tetraploidy induced by somaclonal variation (Nonaka et al., 2023). In contrast, the present findings demonstrate that the iPB-RNP approach effectively avoids cell culture-associated complications, enabling the production of melons with extended shelf-life while maintaining normal morphology and growth characteristics.

**FIGURE 3:**
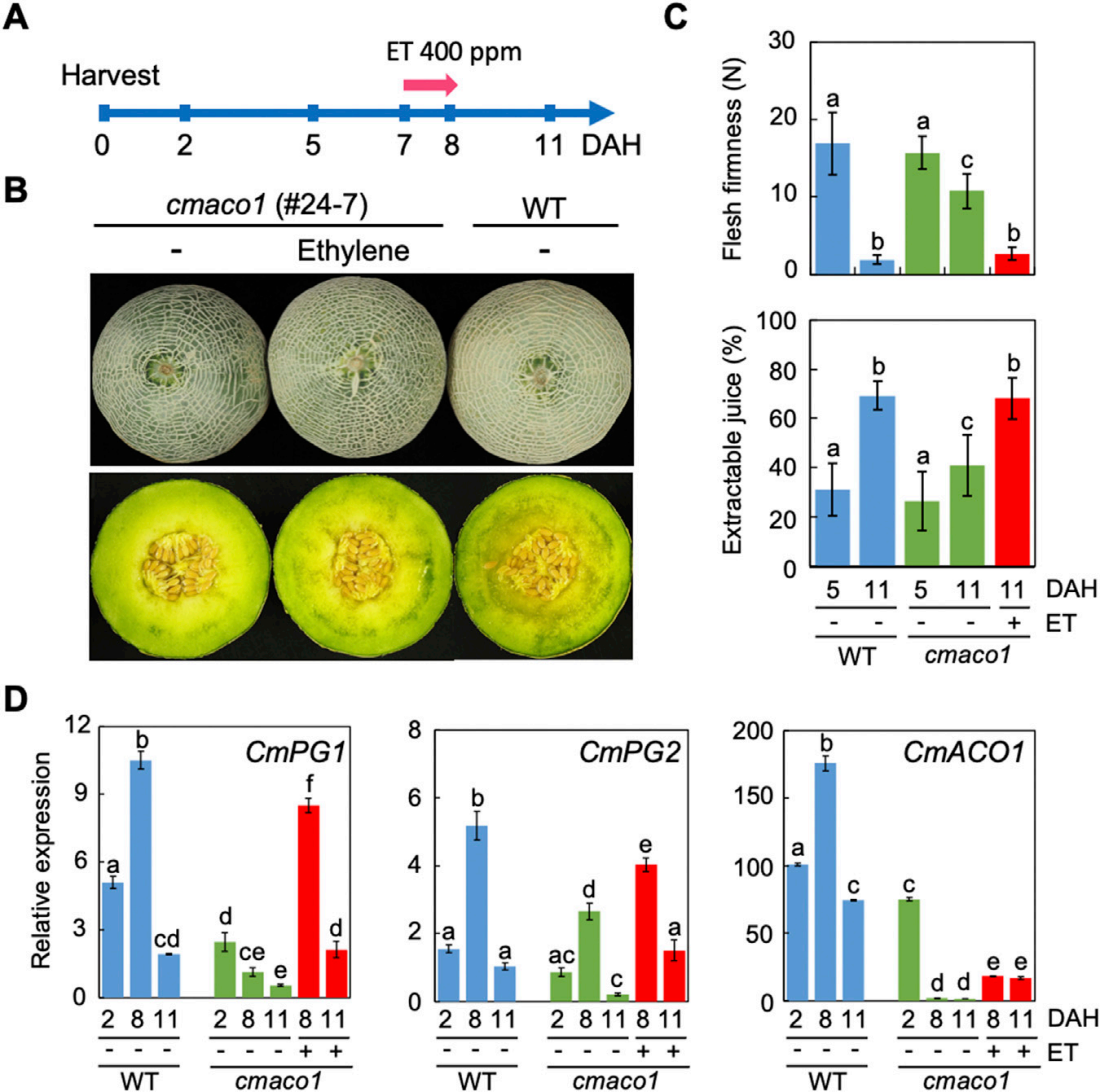
Effect of exogenous ethylene on the *cmaco1* fruit. **(A)** Schematic representation of the experimental design for ethylene treatment of fruits. **(B)** Appearance and longitudinal section of the *cmaco1* fruit 3 days after removal from ethylene exposure following treatment with 400 ppm ethylene. **(C)** Flesh firmness and extractable juice content of the *cmaco1* fruit in the presence or absence of exogenous ethylene. Different lowercase letters indicate significant differences based on Tukey’s honestly significant difference (HSD) test (*P* < 0.05). Means are plotted ±SD (n = 24, three biological replicates with eight measurement points per fruit for flesh firmness; n = 18, three biological replicates with six measurement points per fruit for extractable juice content). **(D)** Relative expression levels of *CmPG1*, *CmPG2*, and *CmACO1* in the mesocarp of *cmaco1* fruit in the presence or absence of exogenous ethylene. The expression level of *CmACO1* in WT at 2 DAH was set to 100. Different lowercase letters indicate significant differences based on Tukey’s honestly significant difference (HSD) test (*P* < 0.05). Means are plotted ±SD from three biological replicates. DAH, Days after harvest; ET, Ethylene; WT, wild-type.

It is commercially important for delayed-ripening melons to retain the ability to respond to exogenous ethylene to initiate ripening. To determine whether genome-edited *cmaco1* fruits are capable of ripening under exogenous ethylene exposure, a short-term ethylene treatment was conducted. At 7 days after harvest (DAH), *cmaco1* fruits were treated with a high concentration of ethylene (400 ppm) for 24 h (Figure 3A). A short-term treatment was selected to avoid the complications associated with continuous ethylene exposure, which requires precise control of ethylene concentration and periodic ventilation to prevent oxygen depletion (Guis et al., 1997; Nishiyama et al., 2007). Three days following the ethylene treatment (11 DAH), a slightly softened flesh texture was observed in the cross-section of the *cmaco1* fruit, indicating that ripening had progressed (Figure 3B). In contrast, *cmaco1* fruit not treated with ethylene retained a firm flesh texture even at 11 DAH (Figure 3B). Because flesh firmness and juice content are key indicators of fruit ripening (Chen et al., 2023), these parameters were measured in *cmaco1* fruits before and after ethylene treatment. In wild-type (WT) fruits, a significant decrease in flesh firmness and an increase in extractable juice content were observed at 11 DAH compared to 5 DAH (Figure 3C). At 5 DAH, *cmaco1* fruits displayed similar levels of firmness and juice content as WT, suggesting minimal ripening activity at this stage. However, at 11 DAH, *cmaco1* fruits exhibited markedly firmer flesh and lower juice content than WT (Figure 3C). Upon ethylene treatment, these values in *cmaco1* fruits shifted to levels comparable to those of fully ripened WT fruit at 11 DAH (Figure 3C). Previous studies reported that continuous ethylene exposure (50 ppm for 4 or 7 days) reversed the delayed-ripening phenotype of *CmACO1* antisense melons (Ayub et al., 1996; Guis et al., 1997). The present data demonstrate that a short, high-concentration ethylene treatment can effectively induce ripening in the *cmaco1* knockout mutant.

The effect of exogenous ethylene on the expression of fruit softening-related genes, *Polygalacturonase1 (CmPG1)* and *Polygalacturonase2 (CmPG2)*, was analyzed in mesocarp tissue. Quantitative PCR (qPCR) analysis revealed that in WT fruit, *CmPG1* and *CmPG2* expression increased during ripening and declined by 11 DAH, when the fruit was fully softened (Figures 3B,D). In the *cmaco1* mutant, *CmPG1* expression was significantly reduced but was restored to WT levels following ethylene treatment (Figure 3D), indicating that *CmPG1* expression is regulated by ethylene during ripening. Similarly, *CmPG2* expression was also reduced in the *cmaco1* mutant and restored by exogenous ethylene. Transient expression of *CmPG1* and *CmPG2* during ripening has been previously reported using gel-blot analysis (Hadfield et al., 1998; Nishiyama et al., 2007). The present qPCR analysis further revealed that *CmPG1* expression was approximately twice as high as *CmPG2* and that both genes were upregulated during ripening. These results suggest that ethylene-induced upregulation of *CmPG* genes contributes to flesh softening in ethylene-treated *cmaco1* fruits.

To evaluate the expression of *CmACO* family genes (*CmACO1–CmACO5*) in mesocarp tissue, it was found that *CmACO1* exhibited the highest and predominant expression in WT during fruit ripening (Supplementary Figure S3), consistent with the conclusion that postharvest ethylene production in melon is primarily mediated by *CmACO1* (Nonaka et al., 2023). In *cmaco1* mutants, only basal levels of *CmACO2–CmACO5* transcripts were detected, both with and without ethylene treatment, and these levels were substantially lower than *CmACO1* expression in WT (Supplementary Figure S3). These findings suggest that fruit ripening in *cmaco1* is triggered by exogenous ethylene rather than endogenous ethylene synthesized via alternative *CmACO* genes. Expression of *CmACO1* in *cmaco1* mutants was slightly lower than that in WT at 2 DAH and dramatically decreased after 8 DAH (Figure 3D). Given that the *cmaco1* mutant harbors a premature stop codon within the *CmACO1* gene (Figure 2G), it is likely that aberrant *CmACO1* transcripts are degraded via nonsense-mediated mRNA decay (Cai et al., 2018) once transcript levels reach a certain threshold.

In conclusion, a non-culture, DNA-free genome editing technique was successfully developed for melon using the iPB-RNP platform. The efficiency of producing genome-edited E_1_ plants from bombarded SAMs was 1.31% for *CmGAD1* and 1.32% for *CmACO1* (Figures 1G, 2F), comparable to efficiencies reported for wheat, barley, and soybean (Kumagai et al., 2022; Kuwabara et al., 2024; Tezuka et al., 2024). As the method eliminates the need for cell culture, it is expected to overcome limitations inherent to traditional transformation approaches, such as genotype dependency and somaclonal variation (Nuñez-Palenius et al., 2008). The successful generation of an extended shelf-life melon line in this study highlights the broad potential of this technique for application in commercial melon breeding and across diverse species within the Cucurbitaceae family.

## Data Availability

The raw data supporting the conclusions of this article will be made available by the authors, without undue reservation.
