# Supplementary material for: A long shelf-life melon created via CRISPR/Cas9 RNP-based in planta genome editing
Source: Front Genome Ed. 2025 Jun 18;7:1623097. doi: 10.3389/fgeed.2025.1623097 (PMC12213888; doi:10.3389/fgeed.2025.1623097)
Supplement: Supplementary file 1 [file Supplementaryfile1.docx]

Supplementary Material

**Supplementary Figure S1.** Microprojectile-mediated GFP gene transfer to melon SAMs. (A) ﻿Bright field and fluorescence images of plants 16 h after bombardment. SAMs are indicated by arrows. (B) Microprojectile-mediated delivery efficiency of GFP plasmid in melon. ﻿

**Supplementary Figure S2.** Schematic representation of the CmGAD1 gene with gRNA design.

**Supplementary Figure S3.** Expression analyses of *CmACO* genes in mesocarp from the *cmaco1*. (A) Relative expression levels of *CmACO1*-*CmACO5* in the presence or absence of exogenous ethylene (400ppm). (B) The enlarged view of *CmACO2*-*CmACO5* expression in A. The expression level of *CmACO1* in WT at 2 DAH was set to a value of 100. Different lowercase letters indicate significant differences by Tukey’s honestly significant difference (HSD) test (*P* < 0.05). Values are means ±SD from three biological replicates. DAH, Days after harvest; ET, Ethylene; WT, Wild-type.

**Supplementary Table S1.** Sequences of the primers used in this study.

**Supplementary Table S2.** ﻿Guide RNA target sites.

**Supplementary Table S3.** ﻿﻿Chemically synthesized guide RNA used in this study
